# Re-expression of ARHI (DIRAS3) induces autophagy in breast cancer cells and enhances the inhibitory effect of paclitaxel

**DOI:** 10.1186/1471-2407-11-22

**Published:** 2011-01-19

**Authors:** Chun-Fang Zou, Luoqi Jia, Hongyan Jin, Ming Yao, Naiqing Zhao, Jin Huan, Zhen Lu, Robert C Bast, Youji Feng, Yinhua Yu

**Affiliations:** 1 Department of Gynecology, Obstetrics and Gynecology Hospital of Fudan University, Shanghai 200011, PR China; 2 Departmen of Laboratory Pathology, Shanghai Cancer Institute, Shanghai 200032, PR China; 3 Department of Bioinformatics, Shanghai Medical College of Fudan University, Shanghai 200032, PR China; 4 Department of Experimental Therapeutics, The University of Texas, M.D. Anderson Cancer Center, Houston, Texas 77030, USA; 5 Department of Obstetrics and Gynecology, Shanghai First People's Hospital of Shanghai Jiao Tong University, Shanghai 200080, PR China

## Abstract

**Background:**

*ARHI *is a Ras-related imprinted gene that inhibits cancer cell growth and motility. ARHI is downregulated in the majority of breast cancers, and loss of its expression is associated with its progression from ductal carcinoma *in situ *(DCIS) to invasive disease. In ovarian cancer, re-expression of ARHI induces autophagy and leads to autophagic death in cell culture; however, ARHI re-expression enables ovarian cancer cells to remain dormant when they are grown in mice as xenografts. The purpose of this study is to examine whether ARHI induces autophagy in breast cancer cells and to evaluate the effects of ARHI gene re-expression in combination with paclitaxel.

**Methods:**

Re-expression of ARHI was achieved by transfection, by treatment with trichostatin A (TSA) or by a combination of TSA and 5-aza-2'-deoxycytidine (DAC) in breast cancer cell cultures and by liposomal delivery of ARHI in breast tumor xenografts.

**Results:**

ARHI re-expression induces autophagy in breast cancer cells, and ARHI is essential for the induction of autophagy. When ARHI was re-expressed in breast cancer cells treated with paclitaxel, the growth inhibitory effect of paclitaxel was enhanced in both the cell culture and the xenografts. Although paclitaxel alone did not induce autophagy in breast cancer cells, it enhanced ARHI-induced autophagy. Conversely, ARHI re-expression promoted paclitaxel-induced apoptosis and G2/M cell cycle arrest.

**Conclusions:**

ARHI re-expression induces autophagic cell death in breast cancer cells and enhances the inhibitory effects of paclitaxel by promoting autophagy, apoptosis, and G2/M cell cycle arrest.

## Background

Breast cancer remains a leading cause of morbidity and mortality in women [1]. Decreasing the recurrence and progression of breast cancer remains a challenge worldwide. Paclitaxel, a naturally occurring anti-microtubule agent that causes mitotic arrest, induces apoptosis in a variety of tumor types [2]. In multiple clinical trials, paclitaxel significantly improved the rates of those who became disease-free and the overall survival rate in breast cancer patients [3-6]. It has become an integral component of first-line adjuvant chemotherapy [7]. Despite the improved efficacy of adjuvant regimens, the recurrence of metastatic disease still contributes to the deaths of hundreds of thousands of women worldwide annually. New approaches are needed to improve the efficacy of paclitaxel.

ARHI encodes a small GTP-binding protein belonging to the *Ras/Rap *superfamily, which has the characteristics of a tumor suppressor gene in ovarian and breast cancers, despite sharing 54-59% homology with Ras proto-oncogenes [8]. ARHI is expressed in normal breast epithelial cells, but it is dramatically downregulated in more than 70% of breast cancers. Loss of ARHI expression has been linked to tumor progression from *in situ *to invasive cancer [9].

*ARHI *is one of ~70 human genes known to be imprinted. The maternal copy is silenced early in embryonic development, and ARHI is expressed only from the paternal allele in all normal cells. As an imprinted gene, loss of ARHI function can be achieved with a "single hit" during carcinogenesis. In cancers tested to date, no mutations of *ARHI *have been found in the coding and promoter regions, but loss of ARHI expression can occur through the loss of heterozygosity, which is observed in 40% of breast cancers [10]. Epigenetic regulation also occurs through downregulation of the paternal allele by DNA hypermethylation, histone deacetylation, and transcriptional regulation. Our previous work has demonstrated that transcriptional repressor complexes, including DNA methyltransferase, histone deacetylase (HDAC) and other repressors, inactivate the *ARHI *gene, and ARHI expression is restored by treatment with the demethylating agent 5-aza-2'-deoxycytidine (DAC) and the HDAC inhibitor trichostatin A (TSA) [11,12].

Our recent findings have shown that re-expression of ARHI in ovarian cancer cell lines induces autophagy and leads to autophagic death in cell culture. When ARHI is upregulated in human xenografts, autophagy is induced. However, cancer cells do not die; they remain dormant and grow promptly when ARHI levels are reduced. Inhibition of autophagy with chloroquine inhibits the outgrowth of dormant cells, which is consistent with the possibility that ARHI-induced autophagy facilitates the survival of dormant cancer cells [13]. Whether re-expression of ARHI can induce autophagy in breast cancer cells has not been previously studied. Similarly, little is known regarding the role of autophagy in the response to cytotoxic chemotherapy. Our present study examines whether ARHI can induce autophagy in breast cancer cells and evaluates the effect of ARHI re-expression on the sensitivity of breast cancer cells to paclitaxel.

## Methods

### Cell culture and reagents

The human breast cancer cell lines SKBr3 and MDA-MB-231 were maintained as described previously [12]. TSA, DAC, rapamycin and paclitaxel were purchased from Sigma (St. Louis, MO). In cell culture, paclitaxel was used at concentrations of 2 nM-4 nM, DAC was used at a concentration of 800 nM, and TSA was used at concentrations of 40 nM-200 nM, depending on the particular cell lines and experiments. The antibodies against ARHI were generated in our laboratory [14]. The antibodies against myosin-associated protein light chain 3 (MAP-LC3), pAKT, AKT, pmTOR, and mTOR were purchased from Cell Signaling Technology (Danvers, MA), the anti-GAPDH antibody was purchased from Jingmei Bio Co. (Shanghai, China), and the anti-β-actin antibody was purchased from Sigma. The siRNAs were purchased from Dharmacon Research (Lafayette, CO).

### Cell proliferation assay

Sulforhodamine B (SRB) was used to detect the effect of drugs on the proliferation of breast cancer cells. The cells were seeded in 96-well plates and incubated with paclitaxel, TSA, or a combination of paclitaxel and TSA for three days. The culture medium was changed daily to maintain the desired concentration of drugs. After 72 hours, 50 μl of 30% trichloroacetic acid was added for 60 min at 4°C. After washing and drying the plate, 100 μl of 0.4% SRB was added for 30 min. Next, the plate was rinsed with 0.1% acetic acid and air dried, and 100 μl of Tris base (10 mmol/L) was added before shaking the plate for 5 min. The SRB value was measured at a wavelength of 570 nm. The experiment was performed in sextuplicate and repeated three times.

### Real-time RT-PCR

The total cellular RNA was extracted using Trizol (Invitrogen, Carlsbad, CA). The first strand cDNA synthesis was carried out with a RevertAid™ first strand cDNA synthesis kit (MBI Fermentas Co., Lithuania) using 2 μg of total RNA and oligo(dT)_12-18 _primers. The quantitative PCR reactions included 2 μl of cDNA and 10 μl of SYBR Green Master Mix (TaKaRa Bio Inc., Dalian, China) with a pair of primers. The reactions were monitored on the ABI PRISM 7000 sequence system (Applied Biosystems Co., Foster City, CA) as described in the manufacturer's protocol. The levels of *ARHI *mRNA were calculated using the equation 2^-ΔΔCT ^and normalized to human *GAPDH *mRNA levels. The primer sequences for real-time quantitative PCR of *ARHI *and *GAPDH *were designed by Sengon Bio Co. (Shanghai, China). The specific primer sequences for *ARHI *were as follows: forward, 5'-TCCGAGCAGCGCATTTGT-3' and reverse, 5'-GGAGCCAAAGCTGGCGTTAC-3'. Primer sequences for *GAPDH *were as follows: forward, 5'-GCACCGTCAAGGCTGAGAAC-3' and reverse, 5'-TGGTGAAGACGCCAGTGGA-3'.

### Flow cytometry analysis of acidic vesicular organelles (AVO)

The cells (1-5 × 10^5^/well) were seeded in six-well plates and incubated overnight. The control and treated cells were stained with acridine orange (Sigma) at a final concentration of 1 μg/ml for a period of 15 min at 37°C while avoiding light. After washing twice with PBS, the cells were trypsinized, suspended in PBS containing 1% FBS and analyzed immediately. The fluorescence emission was measured by flow cytometry (FACS Calibur) (BD, Franklin Lakes, NJ) through the FL1/FL3 channels using CellQuest 7.0 software (Beckman Coulter Co., CA).

### Detection of autophagy with LC3 localization

The SKBr3 cells were co-transfected with pcDNA3-ARHI and GFP-LC3 (provided by N. Mizushima and T. Yoshimori, National Institute for Basic Biology, Okazaki, Japan) using Lipofectamine™ 2000 (Invitrogen); a pcDNA3 vector was used as a control. pcDNA3-ARHI was constructed by inserting ARHI cDNA into a pcDNAS3 vector as previous reported [8]. The cells transfected with GFP-LC3 were also treated with rapamycin (50 nM) as a positive control to induce autophagy. Forty-eight hours after transfection, the cells were fixed with 4% paraformaldehyde for 15 min in the dark, then washed and examined under fluorescence microscopy.

### siRNA transfection

The ARHI siRNAs were a mixture of four designed siRNAs; the efficiency of the knock-down was confirmed by the manufacturer (Dharmacon Research, Lafayette, CO). SKBr3 cells were transfected with the control or the ARHI siRNAs, using the Transfectin #4 reagent (Dharmacon Research), as previously reported [13]. Briefly, a mixture of siRNAs (100 nM final concentration) and transfection reagents were incubated for 20 min at room temperature. This mixture was then added to the cells. After 48 hrs of incubation with the siRNAs and the transfection reagents, the cells were harvested for the measurement of protein and mRNA expression. The drugs were added 24 hrs before harvesting. For experiments with serial transfections, cells were transfected with the siRNAs, using the Transfectin #4 reagent on day one and with additional GFP-LC3 plasmid using Lipofectamine 2000 on day two. The cells were harvested for the measurement of protein expression or fixed for fluorescence microscopy analysis 24 hrs after the final transfection.

### Cell cycle and apoptosis analysis

The breast cancer cells (1-5 × 10^5^/well) were plated and incubated overnight. The control and treated cells were trypsinized, collected in PBS and fixed on ice with 1% paraformaldehyde, followed by 70% cold ethanol. After treatment with 10 μg/ml RNase, the cells were stained with 50 μg/ml propidium iodide (PI, Sigma) for 15 min at room temperature for cell cycle analysis. The apoptotic cells were detected with Annexin V/PI double staining. The cells were trypsinized and stained with Annexin V and PI following the manufacturer's instructions for the Vybrant ^® ^Apoptosis Assay Kit (Invitrogen). The stained cells were analyzed by flow cytometry. Cell cycle information was acquired with CellQuest 7.0 software, and the results were analyzed further using ModFit3.0 software.

### Western blot analysis

The western blot analysis was performed as previously described [14]. Briefly, proteins from total cell lysates were separated using 10-15% SDS-PAGE and then transferred to PVDF membranes (Millipore, Billerica, MA). The membranes were blocked, washed, and incubated with specific primary antibodies. The primary antibody incubation was followed by incubation with HRP-conjugated secondary antibodies (Jingmei Bio Co. Shanghai, China). The bands were detected with an enhanced chemiluminescence assay (Pierce Biotechnology, South Haven, MI).

### Transmission Electron Microscopy (TEM)

The tumor specimens were prepared and processed for electron microscopy. Thin sections were observed with a Philips CM120 transmission electron microscope (Philips, Netherlands). The digital images of the micrographs were obtained with an Epson ES-1200S flat bed scanner and Adobe Photoshop version 5.

### Growth of Xenografts in nu/nu Mice

All animal experiments were carried out in accordance with the animal protocol approved by the Institutional Animal Care and Use Committee of the Shanghai Tumor Institute. Although SKBr3 cells have been extensively characterized in vitro, but this cell line failed to be tumorigenic in nude mice. The impact of ARHI re-expression on tumor growth in vivo was evaluated using xenografts of the human breast cancer cell line MDA-MB-231 in immunosuppressed Nu/Nu Balb/c nu/nu mice. The MDA-MB-231 cells (3 × 10^6^) were injected subcutaneously into the mammary fat pad of each mouse. The tumors were measured twice a week and the tumor volumes were calculated using the formula *TV *= (*L *× *W*^2^)/2, where *L *represents the longer diameter and *W *represents the shorter diameter. When palpable tumors had grown to a diameter of 0.3-0.5 cm, the mice were divided into groups of six to eight, with each group receiving a different treatment. For ARHI treatment, the mice were intratumorally injected three times per week for four to six weeks with 5 μg of ARHI expression plasmid or pcDNA3 vector encapsulated within liposomes (Sigma) at a ratio of 1:10 (DNA:liposomes). As a control, the mice were injected with PBS or liposomes only. The mice in the paclitaxel and paclitaxel/ARHI groups received 10 mg/kg of paclitaxel intravenously three times per week during the same interval.

Two days after the last treatment, two mice from each group were sacrificed and the tumors were collected for TEM analysis. The remaining animals were observed until tumors in the control group reached a diameter of ~1.5 cm.

### Statistical analysis

The means ± SD were used for descriptive analyses. ANOVA was used to assess the differences in means among these groups, multiple comparisons between each two groups were done by the LSD test, and the mixed models were performed to assess the differences in means among these groups for the repeated measurement data. The criterion for statistical significance was taken as *p *< 0.05 using a two-tailed *t*-test. The analyses were performed using Stata 8.0 software.

## Results

### Re-expression of ARHI can be achieved in vitro and in vivo

**Figure 1:**
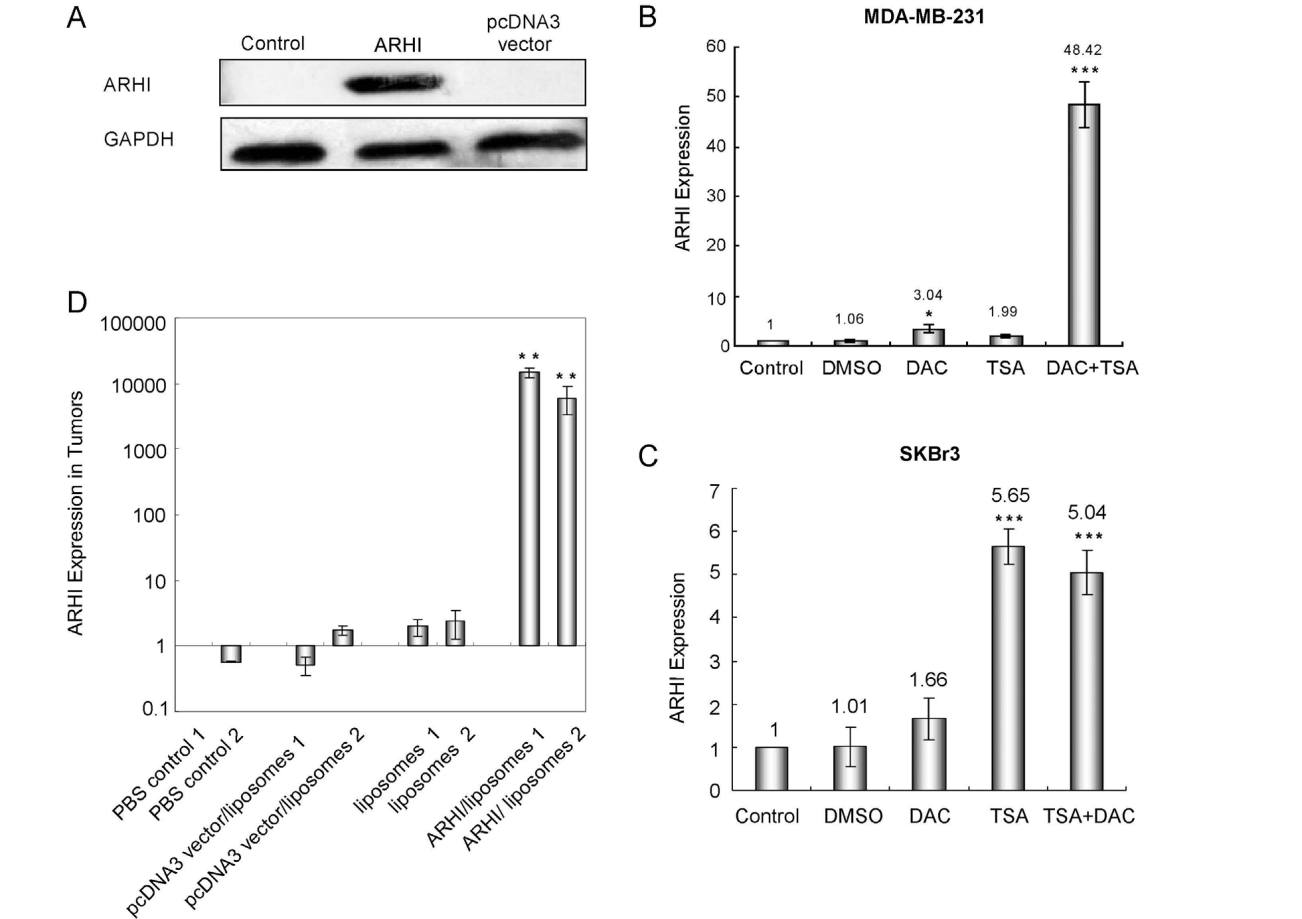
**ARHI re-expression in breast cancer cells**. (A) SKBr3 cells were transfected with pcDNA3-ARHI or pcDNA3 vector constructs. Lipofectamine alone was used as a negative control. The ARHI expression was detected by western blot. (B) MDA-MB-231 cells and (C) SKBr3 cells were treated with DAC, TSA or a combination of DAC and TSA. The untreated cells and cells treated with DMSO diluent were used as negative controls. The ARHI mRNA was detected by real-time quantitative RT-PCR and is presented as the fold change compared to the negative control. *P < 0.05 or ***P < 0.001 compared to the DMSO diluent controls. The data were obtained from three independent experiments. (D) ARHI expression in MDA-MB-231 xenografts was detected by real-time quantitative RT-PCR. It is presented as the fold change compared to the PBS control. Each group included xenografts from two mice, and the data were replicated in three independent experiments. **P < 0.01 compared to pcDNA3 vector/liposomes control.

The breast cancer cell lines SKBr3 and MDA-MB-231, which express little endogenous ARHI, were used to examine the effect of ARHI re-expression in cell culture. ARHI was strongly re-expressed in SKBr3 (Figure 1A) and MDA-MB-231 cells (data not shown) after transfection with an ARHI construct. In MDA-MB-231 cells, in which ARHI was hypermethylated, neither DAC nor TSA alone affected the expression of ARHI; however, robust re-expression of ARHI was detected when cells were treated with a combination of DAC and TSA, demonstrating that these two reagents have synergistic effects (P < 0.001) (Figure 1B). In SKBr3 cells, in which ARHI was partially methylated, DAC did not affect ARHI expression, while TSA alone induced increased expression of ARHI (P < 0.001) (Figure 1C).

We delivered an ARHI expression vector, using liposomes in xenograft models and found that liposomal delivery of ARHI is safe and efficient, permitting multiple injections. Figure 1D shows that ARHI re-expression can be detected at a higher level in the ARHI-liposomes group but not in the PBS control, liposomes, or pcDNA3 vector-liposomes groups.

### ARHI re-expression induces autophagy in breast cancer cells

**Figure 2:**
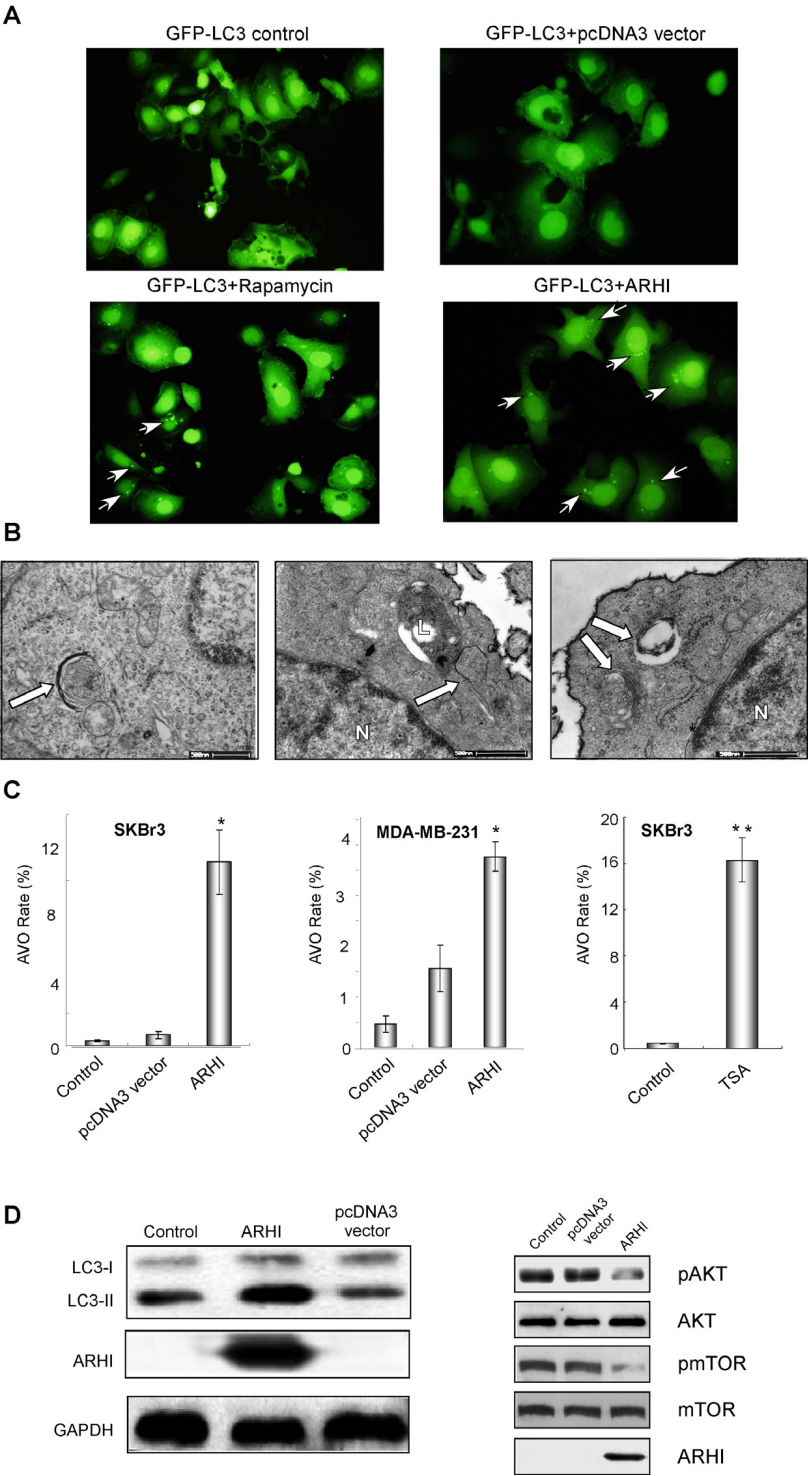
**ARHI induces autophagy in breast cancer cells**. (A) The detection of LC3 punctae. SKBr3 cells were co-transfected with GFP-LC3 and ARHI or pcDNA3 vector. The cells transfected with GFP-LC3 alone and treated with rapamycin served as a positive control. The cells were observed by fluorescence microscopy. The white arrows indicate LC3 punctae. (B) The autophagosomes were detected by TEM in xenografts after treatment with ARHI-liposomes (N, nucleus; L, lysosome). The white arrows indicate autophagosomes. (C) ARHI re-expression increased autophagy induction. SKBr3 cells were transfected with ARHI expression vector (left), MDA-MB-231 cells were transfected with ARHI expression vector (middle) or SKBr3 cells were treated with TSA (right), and flow cytometry-AVO analysis was performed to examine autophagy induction. The data were obtained from three independent experiments. (D) LC3 conversion and signaling. (Left) SKBr3 cells were stably transfected with GFP-LC3 and subsequently transfected with ARHI or pcDNA3 vector constructs. LC3-I and LC3-II were detected by western blot. (Right) SKBr3 cells were transfected with ARHI expression vector. The pcDNA3 vector was used as the negative control. The pAKT, AKT, pmTOR, mTOR and ARHI proteins were detected by western blot.

SKBr3 breast cancer cells were co-transfected with GFP-LC3 and ARHI to detect the effect of ARHI on the formation of autophagic vesicles. Rapamycin-treated SKBr3 cells provided a positive control, whereas the cells transfected with GFP-LC3 alone or GFP-LC3 and pcDNA3 vector served as negative controls. As presented in Figure 2A, the cells transfected with ARHI had an increased number of LC3 punctate spots, which mark autophagosomes and represent the accumulation of a membrane-bound form of LC3 on autophagic vesicles. To further confirm that ARHI re-expression induced autophagy, we performed ultrastructural studies using TEM to detect the induction of autophagosomes in xenografts. As shown in Figure 2B, the tissues that were treated with ARHI-liposomes contained typical scattered double-membrane vacuolar structures. Autophagosomes were not found in xenografts treated with PBS, liposomes or pcDNA3 vector-liposomes.

When ARHI was activated by transfection (Figure 2C left, middle) or by drugs (TSA, Figure 2C right), flow cytometry-AVO analysis showed the autophagy rate was greatly increased. Expression of ARHI increased the conversion of GFP-LC3-I to GFP-LC3-II (Figure 2D left), and ARHI re-expression decreased the phosphorylation of AKT and mTOR, which are known inhibitors of autophagy (Figure 2D right).

### ARHI is required for the induction of autophagy

**Figure 3:**
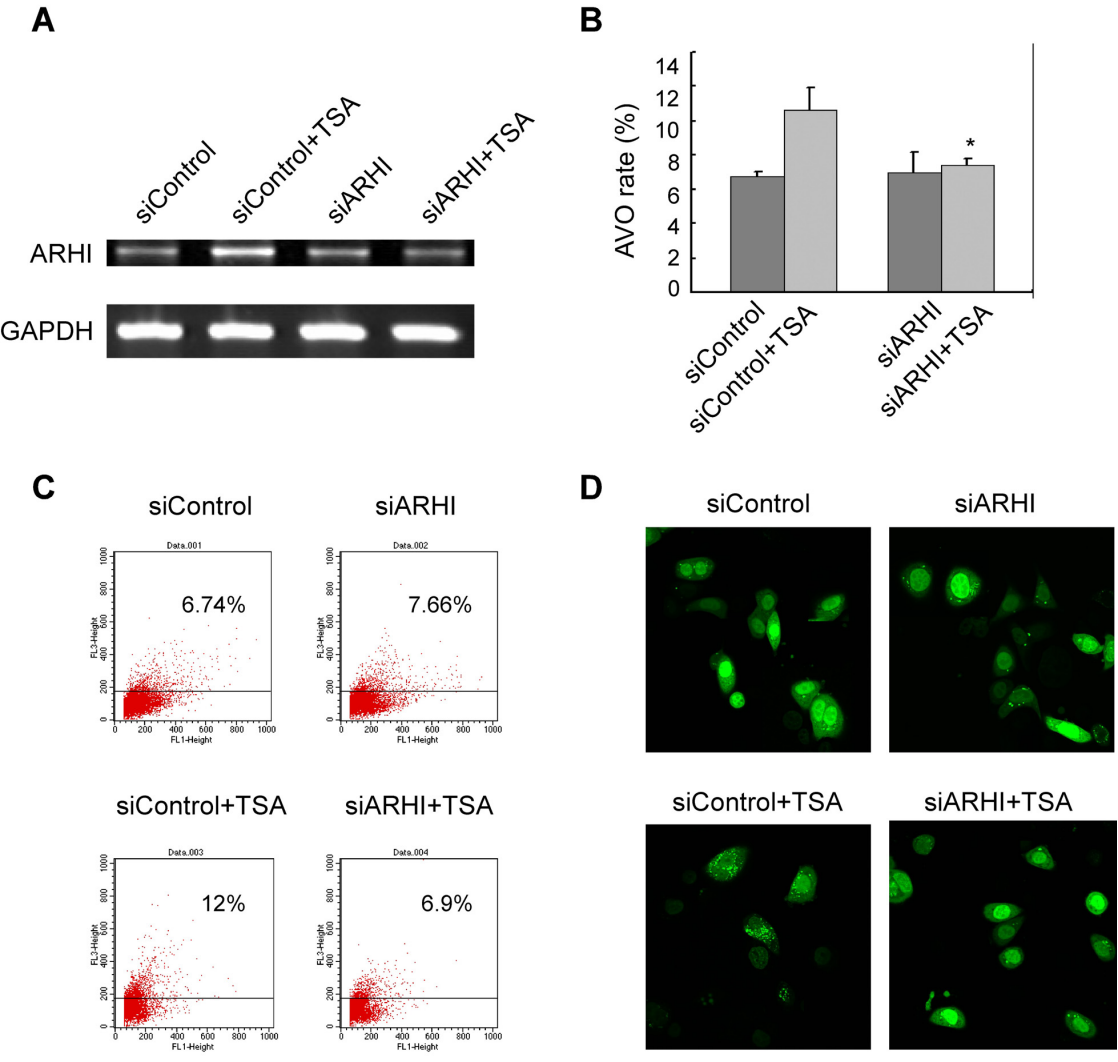
**ARHI siRNA blocks TSA-mediated autophagy induction**. (A) ARHI expression was knocking-down by siRNA. SKBr3 cells were treated with TSA and siRNA-ARHI (siARHI). siRNA-control (siControl) was used as the negative control. ARHI expression was detected by real-time quantitative RT-PCR. (B) TSA-mediated autophagy was blocked by ARHI siRNA. SKBr3 cells were treated with siControl or siARHI only, TSA+siControl or TSA+siARHI. Autophagy was detected by Flow cytometry-AVO analysis. The experiments were repeated three times, *p < 0.05, comparing with TSA/siControl. (C) One representive case of Flow cytometry-AVO analysis from (B). (D) Confocal fluorescent imaging of SKBr3 cells. The cells were co-transfected with a GFP-LC3 construct, and treated with siControl or siARHI only, TSA+siControl or TSA+siARHI.

In all probability, treatment with TSA and DAC upregulates many genes in addition to ARHI. To determine the role of ARHI in the induction of autophagy, we used siRNA to knock down ARHI specifically in SKBr3 cells. TSA treatment upregulated the expression of ARHI, but this upregulation did not occur in the cells transfected with siRNA-ARHI (Figure 3A). Using flow cytometry-AVO analysis (Figure 3B, C) and imaging of LC3 punctate spots (Figure 3D), we observed that TSA treatment enhanced autophagy, but transfection with siRNA-ARHI blocked the effects of TSA, demonstrating that ARHI is essential for autophagy induction.

### Re-expression of ARHI enhances the inhibitory effects of paclitaxel on breast cancer cell growth

**Figure 4:**
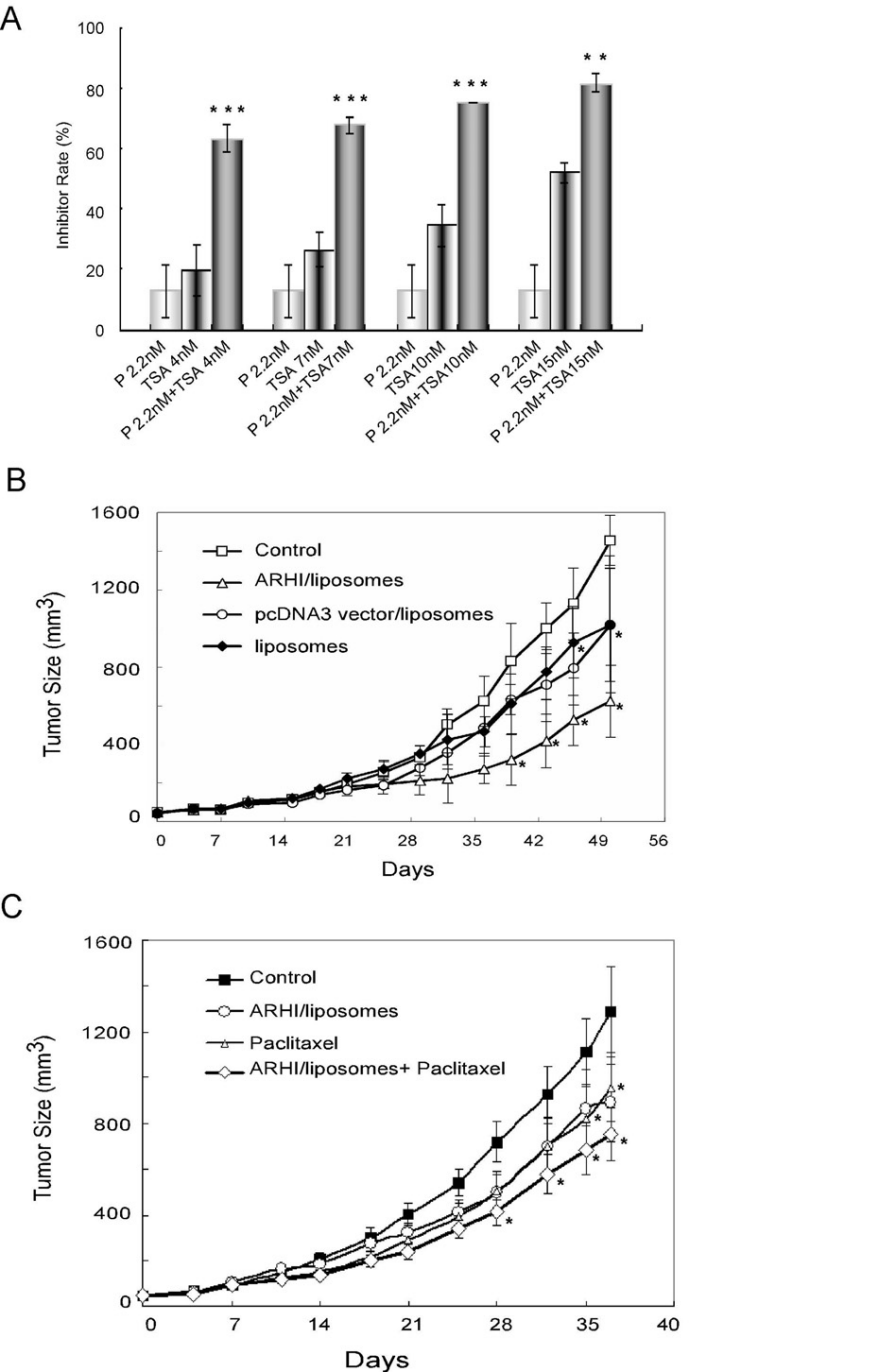
**ARHI re-expression enhances the inhibitory effects of paclitaxel in vitro and in vivo**. (A) SKBr3 cells were treated with paclitaxel (P), TSA (4-15 nM), or a combination of both drugs. The inhibitory rate was detected by SRB assays after three days of culture. **P < 0.01, ***P < 0.001 compared to paclitaxel alone. (B) MDA-MB-231 xenografts were treated with liposomes, pcDNA3 vector/liposomes or ARHI/liposomes. PBS was used as a control. *P < 0.05 compared to control group. (C) MDA-MB-231 xenografts were treated with paclitaxel, ARHI-liposomes or a combination of paclitaxel and ARHI-liposomes. PBS was used as a control. *P < 0.05 compared to control group.

Our recent studies showed that ARHI re-expression can induce autophagic cell death. Under certain conditions, however, ARHI can induce tumor dormancy. To overcome tumor dormancy, we have studied the effects of combined autophagy and apoptosis. Paclitaxel, a cytotoxic drug, can inhibit cancer cell growth by inducing apoptosis and G2/M cell cycle arrest. TSA, an HDAC inhibitor, can activate several tumor suppressor genes, including ARHI, and induce autophagy. In combination assays, TSA greatly enhanced the inhibitory effect of paclitaxel on the growth of SKBr3 cells (P < 0.01 or P < 0.001; Figure 4A). To test if ARHI gene delivery has the same effect, an ARHI expression vector was delivered by intratumoral injection of liposomes. Growth of MDA-MB-231 xenografts was significantly inhibited by ARHI-liposomes, liposomes or pcDNA3 vector-liposomes only showed weaker inhibition (Figure 4B). The tumors treated with a combination of ARHI and paclitaxel grew significantly more slowly (p < 0.05) than the controls, whereas the individual treatments did not significantly inhibit tumor growth (Figure 4C). Thus, ARHI-liposomes enhanced the antitumor activity of paclitaxel.

### Paclitaxel increases ARHI-induced autophagy

**Figure 5:**
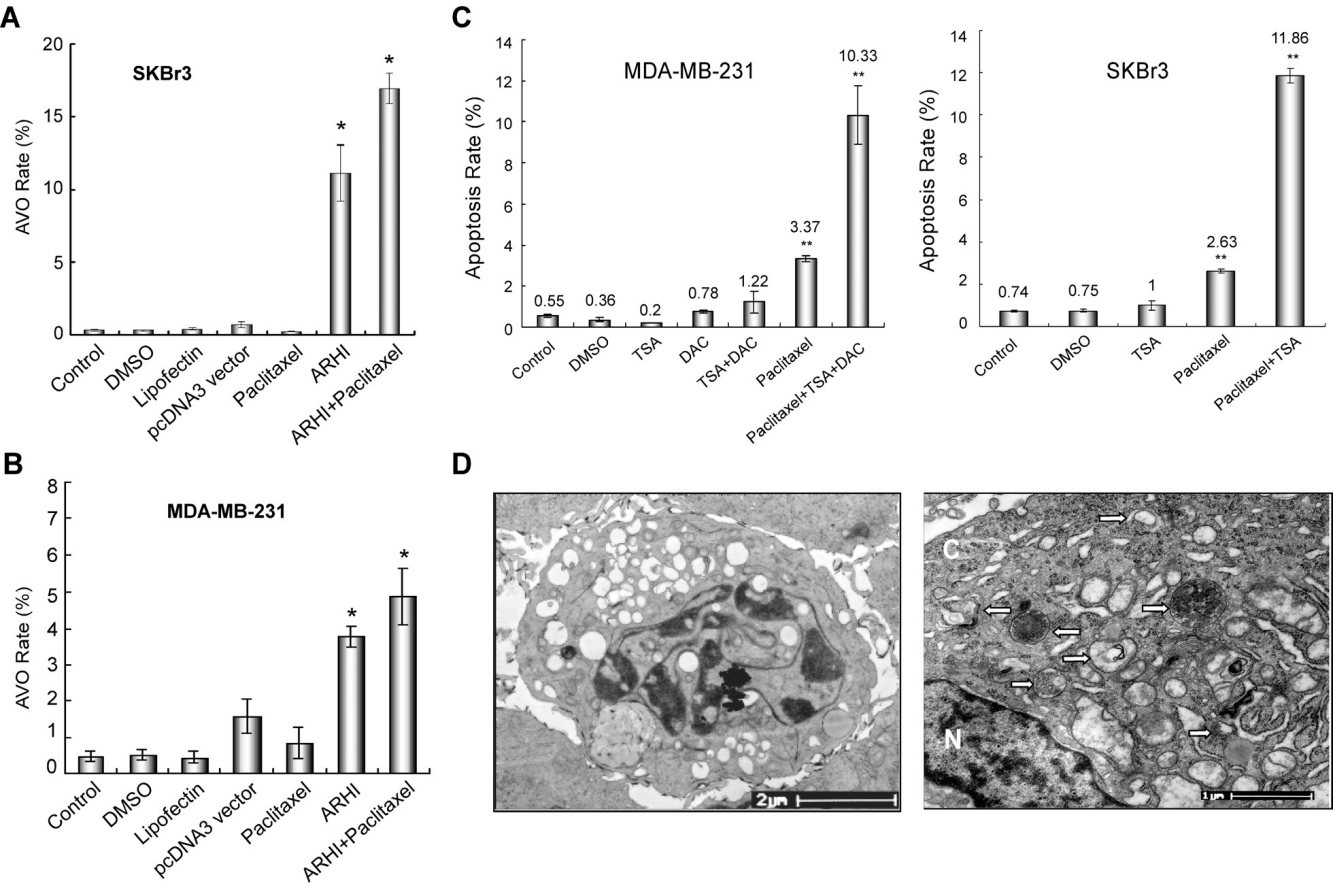
**Autophagy and apoptosis are enhanced by the combination of ARHI re-expression and paclitaxel**. ARHI-induced autophagy is increased by the addition of paclitaxel in SKBr3 (A) and MDA-MB-231 (B) cells. The cells were transfected with pcDNA3-ARHI or pcDNA3 vector constructs. Cells treated with lipofectamine only or DMSO diluent served as negative controls. After 24 hours, paclitaxel was added. Flow cytometry-AVO analysis was performed after 48 hours. *P < 0.05 compared to control. The data were obtained from three independent experiments. (C) Paclitaxel-induced apoptosis is increased by ARHI re-expression. MDA-MB-231 cells or SKBr3 cells were treated with paclitaxel, DAC, TSA, or a combination. The rate of apoptosis was analyzed by annexin-flow cytometry after 48 hours. **P < 0.01 compared to control. The data were obtained from three independent experiments. (D) Both apoptotic cells (left) and autophagic cells (right) were detected by TEM in xenograft tumors that received paclitaxel/ARHI combination treatment.

While paclitaxel alone did not induce autophagy in either MDA-MB-231 or SKBr3 cells, flow cytometry-AVO analysis showed that paclitaxel treatment enhanced ARHI-induced autophagy (Figure 5A, B). ARHI re-expression was performed by transfection (Figure 5A, B) or by TSA treatment (data not shown).

### ARHI re-expression promotes paclitaxel-induced apoptosis

Apoptosis was measured using an annexin V/PI double staining method and flow cytometry analysis. As epigenetic regulators, DAC and TSA can activate several tumor suppressor genes, including ARHI. When DAC and TSA were combined with paclitaxel in MDA-MB-231 cells, or TSA was combined with paclitaxel in SKBr3 cells, respectively (Figure 5C), the number of apoptotic cells significantly increased. In the xenograft model, while autophagosomes were seen in TEM images of the tissues from the mice treated with ARHI-liposomes (Figure 2D), few apoptotic cells were seen in this group. Only classic apoptotic cells with characteristic chromatin condensation and no autophagosomes were seen in tissues from mice treated with paclitaxel. Few autophagic or apoptotic cells were found in the tissues from control mice. Significantly more apoptotic and autophagic cells were found in the tissues from the mice treated with a combination of ARHI and paclitaxel (Figure 5D).

### ARHI re-expression increases paclitaxel-induced G2/M cell cycle arrest

**Figure 6:**
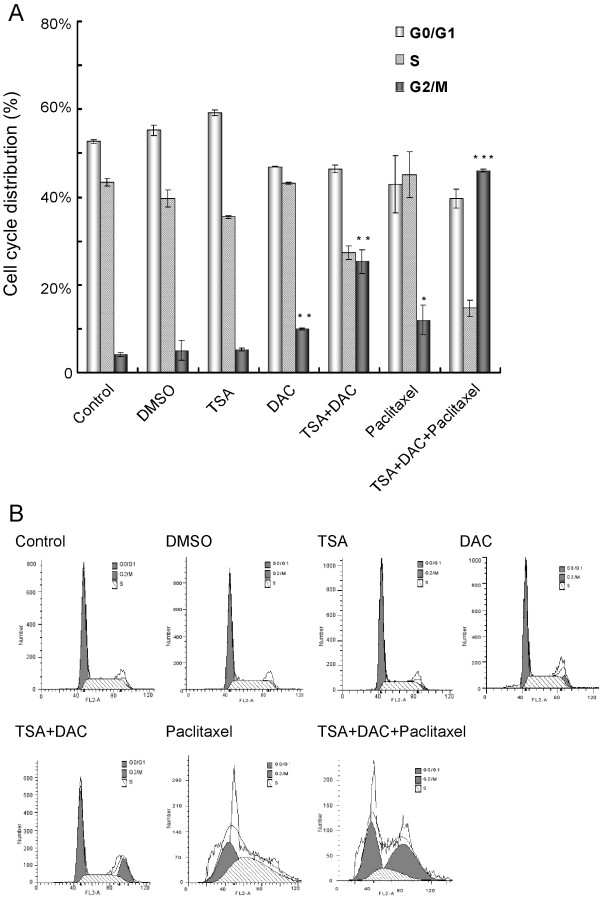
**G2/M cell cycle arrest rate is increased in cells treated with a combination of TSA/DAC and paclitaxel**. (A) MDA-MB-231 cells were treated with paclitaxel, DAC, TSA, or a combination. The cell cycle status was detected by PI staining and flow cytometry analysis 48 hours after treatment. The data were obtained from three independent experiments. *P < 0.05, **P < 0.01, ***P < 0.001 compared to control. (B) Flow cytometry data from one representative experiment.

Paclitaxel is known to induce cell cycle arrest at the G2/M phase. When MDA-MB-231 cells were treated with DAC/TSA in combination with paclitaxel, the frequency of cells arrested at the G2/M phase was dramatically increased (Table 1, Figure 6) compared to the cells treated with paclitaxel alone. Similar results were found in the SKBr3 cells (Table 2).

**Table 1:** The Cell Cycle Analysis of MDA-MB-231 Cells

MDA-MB-231	G0/G1 (%)	S (%)	G2/M (%)
Control	52.70 ± 0.52	43.35 ± 0.95	4.05 ± 0.43
DMSO	55.23 ± 1.17	39.69 ± 2.04	5.09 ± 2.31
TSA	59.25 ± 0.64	35.45 ± 0.38	5.30 ± 0.34*
DAC	46.91 ± 0.16	43.11 ± 0.19	9.98 ± 0.29***
DAC+TSA	46.42 ± 0.81	27.30 ± 1.59	25.29 ± 2.65**
Paclitaxel	42.85 ± 6.51	45.13 ± 5.21	12.01 ± 3.43*
DAC+TSA+Paclitaxel	39.67 ± 2.12	14.6 8 ± 1.87	45.99 ± 0.32***

*P < 0.05, **P < 0.01, ***P < 0.001 compared to control. The data were obtained from three independent experiments and are presented as the mean ± SD.

**Table 2:** The Cell Cycle Analysis of SKBr3 Cells

SKBr3	G0/G1 (%)	S (%)	G2/M (%)
Control	64.25 ± 0.19	31.70 ± 0.63	4.05 ± 0.74
DMSO	65.16 ± 1.24	30.64 ± 1.05	4.20 ± 0.69
Paclitaxel	57.35 ± 1.33	34.70 ± 1.39	7.95 ± 1.34*
TSA	56.12 ± 1.11	37.56 ± 1.12	6.32 ± 0.19*
TSA+Paclitaxel	56.42 ± 1.30	34.15 ± 0.33	9.43 ± 1.50*

*P < 0.05 compared to control. The data were obtained from three independent experiments and are presented as the mean ± SD.

## Discussion

Autophagy is a major intracellular pathway for protein degradation, recycling proteins and eliminating aged or damaged organelles. The process begins with the sequestration of cellular organelles and cytoplasm in a double-membrane autophagosome. Autophagosomes then fuse with lysosomes, and the materials inside are degraded to amino acids and fatty acids. Autophagy is rapidly induced when the cells need to eliminate damaged cytoplasmic components or organelles as well as during oxidative stress, infection, ischemia-reperfusion or mitochondrial dysfunction. Some drugs, such as rapamycin, induce autophagy. Recently, we reported that the tumor suppressor ARHI regulates autophagy and tumor dormancy in ovarian cancer cells. Re-expression of ARHI in multiple human ovarian cancer cell lines induces autophagy by blocking PI3K signaling, inhibiting mammalian target of rapamycin (mTOR), and upregulating ATG4. ARHI also colocalizes with MAP-LC3 in autophagosomes. Furthermore, ARHI is required for spontaneous and rapamycin-induced autophagy in normal and malignant cells [12]. The present study further confirms that the re-expression of ARHI induces autophagy in breast cancer cells and enhances the inhibitory effects of paclitaxel. ARHI activation could be used as a new therapeutic strategy in breast cancer.

Although apoptotic cell death in cancer has been intensively studied over the last decade, the role of autophagy in cell death has received less attention. Autophagy has recently emerged as an important mediator of the programmed cell death (PCD) pathway. Under certain circumstances, the inhibition of autophagy can trigger apoptosis, and the upregulation of autophagy protects against the onset of apoptosis [15]. By contrast, autophagy can function as a second mode of PCD (Type II PCD) that is distinct from apoptosis [16]. Some reports suggest that autophagy protects cells from nutrient depletion stress, but paradoxically, excessive autophagy results in cell death. These results also suggest that the induction of autophagy depends on orchestrated interactions between cancer cells and other cells in the tumor microenvironment during the neoplastic process [17]. Moreover, tumorigenesis is associated with the downregulation of autophagy, and genes that mediate autophagy have been shown to be tumor suppressors [18]. In this study, we addressed the question of what happens when autophagy meets apoptosis by combining agents that induce autophagy and apoptosis and observing their interaction [19]. Our studies revealed that TSA and DAC could activate ARHI expression and induces autophagy, but not apoptosis; it is true that this treatment modulated more genes, but we further confirmed that ARHI is required during autophagy induction. When TSA and DAC were combined with paclitaxel, a chemotherapy agent that induces apoptosis, we observed additive inhibitory effects on breast cancer cell growth. Paclitaxel alone did not induce autophagy in these breast cancer cell lines, but an increased fraction of autophagic cells was found when paclitaxel was combined with TSA and DAC. Conversely, apoptosis was not induced when ARHI was activated by TSA and DAC, but the fraction of apoptotic cells was increased when paclitaxel was added.

Whether the induction of autophagy contributes to cell survival or death during apoptosis may relate to the level and duration of autophagy. Low levels of autophagy could promote survival by eliminating damaged intracellular proteins and organelles. This process could increase cell survival by preventing apoptosis. Conversely, excessive autophagy, such as that observed during the induction of ARHI re-expression by TSA and DAC, eventually led to the destruction of essential proteins and organelles beyond a certain threshold, resulting in cell death. In examining various signaling pathways, we found ARHI re-expression mainly inhibited the autophagy-related pathways, such as pAKT and pmTOR (Figure 2), while paclitaxel mainly inhibited the apoptosis-related pathway. When these signaling changes were combined, cancer cell death was hastened. Our studies may thus shed light on a new approach for combining ARHI gene therapy with chemotherapy.

## Conclusions

A tumor suppressor gene, ARHI, induces autophagy in breast cancer cells, and ARHI is essential for the induction of autophagy. When ARHI was re-expressed in breast cancer cells treated with paclitaxel, the growth inhibitory effect of paclitaxel was enhanced by promoting autophagy, apoptosis, and G2/M cell cycle arrest.

## List of Abbreviations Used

DAC: 5-aza-2'-deoxycytidine; TSA: trichostatin A; MAP-LC3: myosin-associated protein light chain 3; TEM: transmission electron microscopy; AVO: acidic vesicular organelles; SRB: sulforhodamine B; HDAC: histone deacetylase; DCIS: ductal carcinoma *in situ*.

## Competing interests

The authors declare that they have no competing interests.

## Authors' contributions

CFZ carried out the autophagy and animal assays and prepared the manuscript. LJ carried out the signaling and FACS analysis. HJ provided support in the expression analysis and helped to prepare the manuscript. MY provided support in the animal studies. NZ and JH performed the statistical analyses. ZL provided support for the autophagy studies. RCB, YF & YY conceived of the study and gave final approval for the manuscript. All authors read and approved the final manuscript.

## Pre-publication history

The pre-publication history for this paper can be accessed here:

http://www.biomedcentral.com/1471-2407/11/22/prepub
